# Transformer-based tool recommendation system in Galaxy

**DOI:** 10.1186/s12859-023-05573-w

**Published:** 2023-11-27

**Authors:** Anup Kumar, Björn Grüning, Rolf Backofen

**Affiliations:** 1 Bioinformatics Group, Department of Computer Science, University of Freiburg, Georges-Koehler-Allee 106, 79110 Freiburg, Germany; 2 Signalling Research Centres BIOSS and CIBSS, University of Freiburg, Schaenzlestr. 18, 79104 Freiburg, Germany

**Keywords:** Galaxy, Tools, Workflows, Artificial intelligence, Transformer, Recommendation system

## Abstract

**Background:**

Galaxy is a web-based open-source platform for scientific analyses. Researchers use thousands of high-quality tools and workflows for their respective analyses in Galaxy. Tool recommender system predicts a collection of tools that can be used to extend an analysis. In this work, a tool recommender system is developed by training a transformer on workflows available on Galaxy Europe and its performance is compared to other neural networks such as recurrent, convolutional and dense neural networks.

**Results:**

The transformer neural network achieves two times faster convergence, has significantly lower model usage (model reconstruction and prediction) time and shows a better generalisation that goes beyond training workflows than the older tool recommender system created using RNN in Galaxy. In addition, the transformer also outperforms CNN and DNN on several key indicators. It achieves a faster convergence time, lower model usage time, and higher quality tool recommendations than CNN. Compared to DNN, it converges faster to a higher precision@k metric (approximately 0.98 by transformer compared to approximately 0.9 by DNN) and shows higher quality tool recommendations.

**Conclusion:**

Our work shows a novel usage of transformers to recommend tools for extending scientific workflows. A more robust tool recommendation model, created using a transformer, having significantly lower usage time than RNN and CNN, higher precision@k than DNN, and higher quality tool recommendations than all three neural networks, will benefit researchers in creating scientifically significant workflows and exploratory data analysis in Galaxy. Additionally, the ability to train faster than all three neural networks imparts more scalability for training on larger datasets consisting of millions of tool sequences. Open-source scripts to create the recommendation model are available under MIT licence at https://github.com/anuprulez/galaxy_tool_recommendation_transformers

**Supplementary Information:**

The online version contains supplementary material available at 10.1186/s12859-023-05573-w.

## Background

A rapid increase in the number of scientific tools performing various tasks in different fields of life sciences makes constructing workflows using these tools more complicated. Assembling such scientific tools into a workflow poses a significant challenge, as the analysis represented by the workflow should incorporate scientifically significant steps and produce reproducible results. To simplify creating workflows, a tool recommender [1] in Galaxy [2] was created using good-quality workflows stored in Galaxy Europe. This recommender system trains a recurrent neural network (RNN) on existing workflows and creates a model that predicts scientific tools at each step of creating workflows. Each step considers the sequence of tools or already created a workflow to recommend tools.

### Comparison to state-of-the-art approaches

In comparison to other workflow recommendation systems such as WINGS [3] and PROPHETS [4] that require explicit annotations in terms of input data types and parameters, and functions of tools, the deep learning-based approach (RNN) requires only sequences of tools for training and creating a recommendation model. In addition, the accuracy of RNN in recommending tools is significantly higher compared to non-neural network-based machine learning algorithms such as ExtraTrees classifier [1]. Another approach that uses collaborative filtering for workflow recommendation achieves low accuracy of 0.83 AUC on a dataset collected from Canadian Open Neuroscience Platform [5]. Even though RNN architecture has been popular for modelling sequential data and achieves high accuracy in workflow recommendation, there are a few drawbacks to such an architecture. First, the training and convergence times of RNN are significantly higher than the transformer’s as it is harder to parallelise mathematical computations because of RNN’s recurrent connections. Larger memory consumption allows training only in small batches of workflows, making it more challenging to train such an architecture as data grows with time. Second, the trained RNN has a considerably larger usage time, the combination of time needed to recreate the model from its saved format and predict using a tool or tool sequence, than the transformer. Third, the prediction performance of RNN suffers for longer sequences, and lastly, the generalisation ability of the transformer is better compared to RNN [6]. Further, the transformer outperforms convolutional (CNN) and dense (DNN) neural networks on various indicators such as convergence speed, model usage time, and quality of tool recommendations (see Results section for detailed comparison). Therefore, the recommendation model is created by training a transformer architecture using workflows created in Galaxy Europe to acquire its advantages.

### Transformers

Several studies successfully used transformers to model sequential data that achieve start-of-the-art outcomes. Bidirectional encoder representations from transformers (BERT) have been vastly used for modelling languages and achieved exceptional results on eleven natural language tasks [7]. DNABERT creates a novel model taking cues from BERT architecture by training on DNA sequences. It is used for many downstream tasks, such as predicting regulatory elements, promoters, splice sites and transcription factor binding sites with high accuracy [8]. ProteinBERT improves the BERT architecture to model protein sequences and achieves excellent results on various tasks such as predicting protein functions and gene ontology (GO) annotations [9]. Transformer uses the attention mechanism [6] to learn representations of sequential data such as natural languages and DNA and protein sequences. With self-attention (see “Self-attention” paragraph), each token in a sequence is assigned a weight that explains its correlation to all other tokens. A token with a larger magnitude of weight is more important than one with a smaller magnitude in prediction tasks such as sequence classification. These weights, represented by real numbers, are collectively used to compute predictions. The architecture of a transformer has several components. The major ones are the encoder and decoder [6]. For sequential data consisting of input and output sequences, the encoder learns the representation of input sequences, and the decoder jointly trains on the input representation by the encoder and the output sequences. In our work, only the encoder part of the transformer is used to create the recommendation model. This model recommends tools for both - a tool and a tool sequence. The architecture of the transformer used for creating the Galaxy tool recommendation system is discussed in the Architecture section.

#### Self-attention

**Fig. 1:**
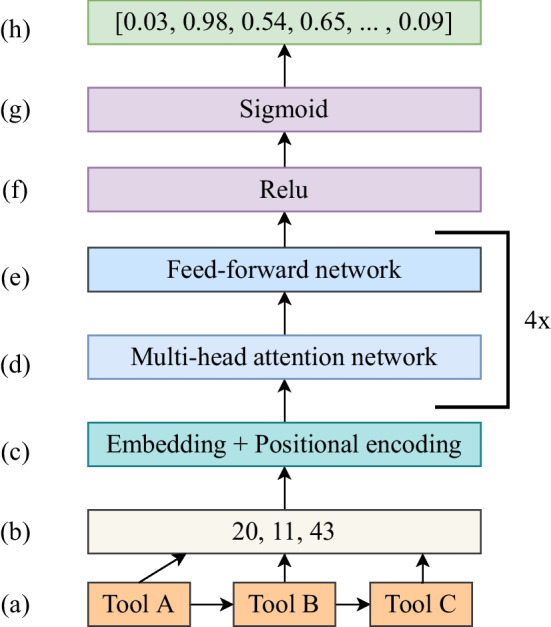
Neural network architecture of transformer used for recommending tools in Galaxy. Figure 1a–b represents how a sequence of tools is transformed into a sequence of integers. Figure 1c–g represents several different neural network layers through which sequences of tools are passed to learn their respective representations and mapping with their respective labels. Figure 1h represents the output in the form of a real-valued vector

Transformer employs the self-attention mechanism to learn the representation of a sequence. With self-attention, each token of a sequence relates to all other tokens of the same sequence but with different magnitudes [6]. Our work uses self-attention to compute the vector representation of each tool sequence that maps with its multi-hot encoded labels to predict tools. For example, in a sequence of three tools in Fig. 1a, we assume that tools A and B are related, but tool A is unrelated to tool C. Therefore, this magnitude of relatedness, also known as attention weights, is higher between tools A and B than between tools A and C. All attention weights belonging to each pair of tokens are utilised in computing a vector representation of the sequence, implying that the representation contains the context of the entire sequence. RNN [10] works differently, which creates a sequence’s representation using the last token and the sequence’s context stored for the last but one token, thereby making it prone to losing context for a longer sequence.

## Implementation

### Data preparation

Galaxy workflows [11] are directed acyclic graphs in which nodes are represented by scientific tools, as shown in Additional file 1. All the workflows used in this work have been collected from Galaxy Europe using a bash script [12]. The workflows extracted from the script are stored in a tabular file and are pre-processed to extract tool sequences. A tool or a tool sequence can connect to multiple tools, which become the tool’s or tool sequence’s labels. There are thousands of tools on Galaxy Europe, and each can connect to many others. Therefore, predicting tools for a tool or a sequence becomes a multi-class, multi-label classification. To represent tool sequences in a manner to be interpreted by any neural network, each tool is assigned a unique integer. This step transforms each sequence of tools into a sequence of integers. A tool or a set of tools can extend an analysis using a sample workflow in Fig. 1a by adding tools to tool C. A tool or the set of tools used to extend the workflow “tool A $\rightarrow$ tool B $\rightarrow$ tool C” become the recommended tools of the “tool A $\rightarrow$ tool B $\rightarrow$ tool C” workflow. The recommended tools for each sequence of tools are represented as multi-hot encoding, as shown in Additional file 2 (part D). The multi-hot encoding of each tool sequence is its label represented as a 1-dimensional vector. The corresponding pairs of tool sequences and their respective labels are used for training the transformer to create a tool recommendation model. Other neural networks, such as RNN, CNN and DNN, also use the same data for training.

#### Data statistics

Approximately 60,000 workflows have been collected from Galaxy Europe, which are published and contain no errors. These workflows are further pre-processed to extract approximately 7,98,000 linear tool sequences, of which 3,53,000 are unique. The longest tool sequence contains 28 tools, the shortest ones have two tools, and the median length is 15. There is a significant variance in the number of occurrences of different tools. For example, text formatting tools such as “cut” and “filter” are present in over 2,00,000 of these tool sequences, while a tool such as “graphlan” [13] occurs only 40 times in these tool sequences. Therefore, to allow the transformer to learn from a similar distribution of sequences of tools, each training batch is sampled to contain labels with similar frequencies. Each tool or a tool sequence can connect to multiple unique tools. Therefore, the unique tool sequences are further pre-processed to create tool sequences that may have more than one tool as labels and are transformed to multi-hot encoded vectors. This further increases the number of unique tool sequences with their respective multi-hot encoded labels to approximately 5,00,000. The number of unique tools to create all used workflows for our work is 2,354.

### Architectures

#### Transformer

The encoder part of the transformer in [6] consists of multiple layers, as shown in Fig. 1. The first is the embedding and positional encoding layers (Fig. 1c). These two layers work together to encode tools, where each tool is represented by a unique integer (Fig. 1b) in a workflow (Fig. 1a). Tools are tokens sequentially arranged in workflows. The encodings of tools contain real-valued vectors that conserve the relative positions of tools in workflows. The embedding and positional encoding layers are implemented using the embedding layers [14]. The encodings of sequences of tools are passed to a composite block consisting of multi-head attention [15] and feed-forward layers (Fig. 1d–e). The term 4x in Fig. 1d–e represents four attention networks working together in parallel. Attention weights learned by each attention head are concatenated. The multi-head attention layer is 128 dimensional, and the feed-forward neural network consists of two dense layers [16] having 128 dimensions each. Collectively, they compute representations of sequences based on self-attention weights. Tools related to one another in tool sequences get higher attention weights. In addition, they capture long-range dependencies among tools and make the trained models more interpretable [6]. In the next step, these representations are passed to a dense layer with 128 dimensions and relu activation [17] (Fig. 1f). The predicted output, computed by a different dense output layer, is a vector of sigmoid [18] scores (Fig. 1h) used to recommend top N tools. The higher the sigmoid score, the higher the probability of a tool being correctly recommended. The number of trainable parameters in the transformer architecture is 9,22,291. To measure the performance of the transformer in recommending tools, its architecture is compared to three different neural network architectures - RNN, CNN and DNN on several key indicators such as convergence time, model usage time and the quality of tool recommendations.

#### RNN

Gated recurrent units (GRU) [19] layers are used to implement RNN architecture [20] to model the sequence of tools. The RNN architecture uses an embedding layer to learn a fixed-size vector representation (128) for each tool, also known as embedding dimensions. The embedding layer is followed by two GRU layers stacked on each other. The dimensions of both the GRU layers are 128. The output of the second (last) GRU layer is passed to an output layer, a dense layer with sigmoid activation, and its dimension equals the number of tools. The total number of trainable parameters of RNN is 2,41,063.

#### CNN

The CNN architecture [21], like RNN, also has an embedding layer to learn the fixed-size vector of each tool. The sequences of tools represented by the embeddings of each tool in sequences are passed to a 2-dimensional convolutional layer [22] having 128 dimensions with a kernel of size 16 x 3 and relu activation. The output of the convolutional layer is passed to the maxpooling2D [23] layer for extracting maximum values from each pool size of 2x2. These values are further passed to a flatten [24] layer that arranges the output from the maxpooling2D layer horizontally. This horizontally arranged output is then passed to a dense layer with a dimension 128 connected to an output layer like RNN. These layers in CNN have 5,772,723 as the total number of trainable parameters.

#### DNN

The DNN architecture [25] consists of only one embedding layer with a dimension of 128 and two dense layers, each having a dimension of 128. The output layer of DNN is the same as RNN and CNN architectures. The number of trainable parameters in DNN is 1,031,603.

### Training parameters

Python 3.9 is used to create scripts stored at GitHub [26] to train the different models such as transformer, RNN, CNN and DNN. All the models are implemented using TensorFlow 2.9 [27] and are trained on a machine with Rocky Linux release 8.5 with approximately 35 CPU cores and over 100 GB memory for 35,000 iterations with a 512 batch size. Adam optimiser [28] minimises the binary cross-entropy [29] loss with a standard learning rate of 0.001. Dropout [30] layers are used in all architectures to minimise overfitting. The degree of dropout used is 0.2. Hyperparameters of the transformer neural network are estimated manually. All the sequences of tools (approximately 500,000) are divided into train and test sets. 80% of the sequences are used for training (approximately 400,000) and the rest for evaluating (approximately 100,000) the trained model. The training time for 35,000 iterations varies for all models. Training the transformer takes approximately 39 h (3.96 s per iteration), RNN takes approximately 43 h (4.4 s per iteration), CNN takes approximately 50 h (5.1 s per iteration), and DNN takes approximately 43 h (4.4 s per iteration). Transformer requires the least time for training compared to RNN, CNN and DNN models.

**Fig. 2:**
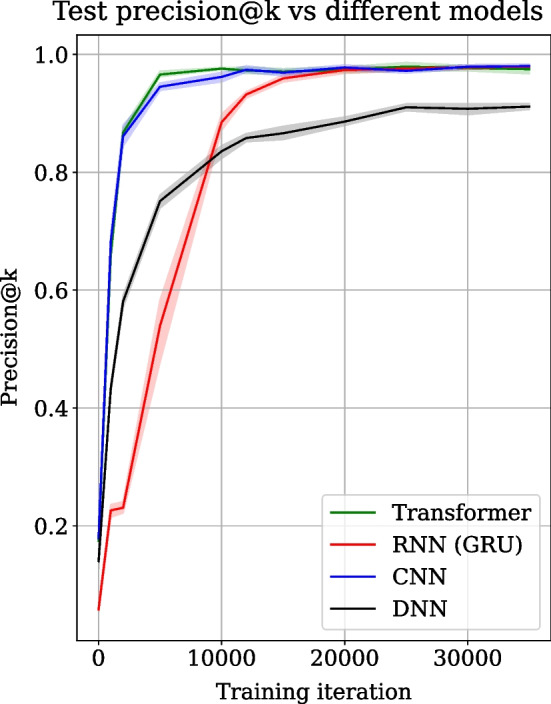
A comparison of precision@k metric, used for evaluating a recommender system, is shown over training iterations for the transformer (green), RNN (red), CNN (blue) and DNN (black) architectures. The precision@k values are averaged over 5 experiment runs for each architecture and are shown as line plots. The shaded regions show the standard deviation across 5 experiment runs

## Results

### Transformer convergence time

Transformer [31], RNN [20], CNN [21] and DNN [25] architectures are trained on the same dataset, and their precision@k score collected over 35,000 training iterations on the test data are compared in Fig. 2. Precision@k is used as the prediction metric and is popular for evaluating recommender systems [32–34]. The precision@k scores are averaged over 5 experiment runs. The shaded regions show the standard deviation over experiment runs. The train and test datasets are randomly created in each run but have the same size. Figure 2 shows the mean precision@k score in green for the transformer, red for RNN, blue for CNN and black for DNN. Figure 2 shows that the transformer converges two times faster than RNN. The transformer, consisting of two embedding layers, a multi-head attention layer, a hidden dense layer and a dense output layer, converges to the mean precision@k score of approximately 0.98 at iteration 10,000. In contrast, RNN, consisting of one embedding layer, two stacked GRU layers and one dense output layer, achieves similar mean precision@k only at iteration 20,000. DNN, consisting of one embedding layer, two hidden dense layers and one dense output layer, also shows slower convergence and a lower precision@k score (0.9). On the other hand, CNN consisting of one embedding layer, one conv2d, one maxpooling2d, one flatten, one hidden dense, and one dense output layer, converges to a similar precision@k value achieved by the transformer but is slightly slower in convergence and stabilises around 12,000 iterations. Further, the transformer achieves a similar mean precision@k score (approximately 0.85) to CNN but slightly worse than RNN for infrequent tools, making the lowest $25\%$ of all tools, as shown in Additional file 3. The performance of DNN for the lowest $25\%$ of all tools is significantly lower (approximately 0.72) compared to other models (Additional file 3). All other parameters of the experiment remain the same.

**Fig. 3:**
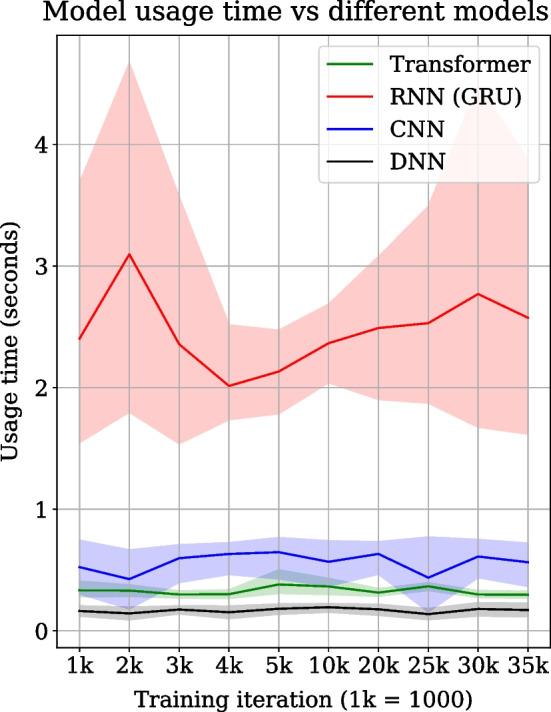
A comparison of the model usage (model reconstruction + prediction) time of transformer (green), RNN (red), CNN (blue) and DNN (black) is shown. The time is averaged over 5 experiment runs, and the shaded region shows the standard deviation across 5 experiment runs

### Transformer usage time

Transformer, RNN, CNN and DNN architectures are compared for their respective model reconstruction and prediction time measured at specific intervals over 35,000 training iterations, as shown in Fig. 3. The transformer’s model usage time (= model reconstruction and prediction) is approximately 0.4 s for a sequence of tools. At the same time, for RNN, it is over 2 s which is approximately four to five times slower than the transformer. CNN is also faster than RNN in model usage time but is slower than the transformer. However, DNN is the fastest, recording the lowest model usage time out of all models. The model usage time is averaged over all the sequences of tools in the test dataset. The line plots show the mean usage time, and shaded regions show the standard deviation over 5 experiment runs (Fig. 3).

**Table 1:** Comparison of recommendations by transformer and RNN for multiple scientific analyses

Scientific analysis	Tool/Tool sequences	Ground truth	Transformer	RNN
1. Variant analysis	snpeff_sars_cov_2 [36]	snpSift_filter, snpSift_extractFields, lofreq_filter, vcf2tsv, multiqc, collapse_dataset, CONVERTER_vcf_to_vcf_bgzip_0 [36]	multiqc, collapse_dataset, CONVERTER_vcf_to_vcf_bgzip_0, snpSift_extractFields, vcf2tsv, snpSift_filter, lofreq_filter, **freebayes**, **mimodd_varcall**, **snpfreqplot**, **gemini_load**, **vcfcombine** [35, 38]	multiqc, collapse_dataset, vcf2tsv, CONVERTER_vcf_to_vcf_bgzip_0, lofreq_filter, snpSift_extractFields, snpSift_filter, **tb_variant_filter**, **mimodd_map**, **vcffilter2** [37, 40]
2. Single-cell	anndata_import [52]	scanpy_filter, anndata_inspect, anndata_manipulate, ucsc_cell_browser, scanpy_inspect, scanpy_filter_cells [52, 53]	scanpy_filter_cells, anndata_inspect, ucsc_cell_browser, scanpy_filter, scanpy_inspect, anndata_manipulate, **scanpy_normalise_data**, **scanpy_plot**, **anndata_ops**, **scanpy_remove_confounders**, **scanpy_integrate_harmony**, **scanpy_normalize**, **scpred_get_feature_space**, **scanpy_find_variable_genes**, **scpred_predict_labels**, **scpred_eigen_decompose** [53, 54]	anndata_manipulate, scanpy_filter, scanpy_filter_cells, ucsc_cell_browser, scanpy_inspect, anndata_inspect, **scanpy_plot**, **scanpy_normalise_data**, **scmap_scmap_cluster**, **scmap_scmap_cell**, **scanpy_filter_genes** [52, 53, 55]
3. Deep learning	keras_train_and_eval [56]	model_prediction, ml_visualization_ex [56]	ml_visualization_ex, model_prediction, **plotly_regression-_performanc_plot**, **sklearn_discriminant_classifier**, **plotly_ml_performance_plots** [56]	model_prediction, ml_visualization_ex [56], **nn_classifier** [56]
4. Prote-omics	mass_spectrometry-_imaging_filtering, cardinal-_preprocessing, cardinal-_segmentations [41]	Filter1	Filter1, **cardinal_spectra_plots**, **cardinal_combine**, **cardinal_mz_images**, **cardinal_classification**, **cardinal_data_exporter**, **cardinal_quality_report**, **maldi_quant_preprocessing**, **cardinal_preprocessing**, **cardinal_segmentations**, **cardinal_filtering**, **mass_spectrometry-_imaging_filtering**, **maldi_quant_peak_detection** [41]	Filter1

### Generalisation beyond training data

Trained transformer and RNN models are used to compare the top 20 recommendations made for tools and sequences of tools belonging to multiple scientific analyses, as shown in Table 1. The ground truth column (third column) shows accurate tools extracted from the training workflows for a tool or a tool sequence in the second column. The fourth and fifth columns show the recommended tools by the transformer and RNN models, respectively. The tools shown in bold are those recommended tools compatible with the respective tool or tool sequence in the second column, but such connections are not available in the training workflows. To elaborate, the recommended tool “freebayes” [35] (shown in the fourth column of the first row of Table 1) can be used on the output datasets produced by “snpeff_sars_cov_2” [36] tool to extend a workflow for variant calling analysis. However, this connection does not exist in the training workflows used to train the transformer model as it is absent from the ground truth recommendation of the “snpeff_sars_cov_2” tool. Similarly, tools such as “mimodd_varcall” [37], “snpfreqplot” [38], “gemini_load” [39] and “vcfcombine” [40] can also be used to extend a scientific analysis after using “snpeff_sars_cov_2” tool but its connections to these recommended tools do not exist in the training workflows. RNN model also recommends three tools beyond the seen training workflows, but the transformer recommends five such tools. Row 4 in Table 1 shows another example from the proteomics field, where both models correctly predict the ground truth recommendations. However, the transformer recommends many other tools that are from the proteomics field and can be used to extend the “mass_spectrometry_imaging_filtering $\rightarrow$ “cardinal_preprocessing $\rightarrow$ cardinal_segmentations” [41] (fourth row of Table 1) workflow. Similar examples of better generalisation of the transformer are found for other scientific analyses, such as single-cell and deep learning, where the sets of high-quality recommendations by the transformer going beyond the training workflows are significantly larger than that by RNN. In conclusion, Table 1 shows that the transformer model generalises better than the RNN model in recommending tools for scientific workflows as it predicts valid recommendations that have not been seen while training. Table 1 is further extended to add tool recommendations by CNN and DNN models and is shown in Additional file 4.

**Fig. 4:**
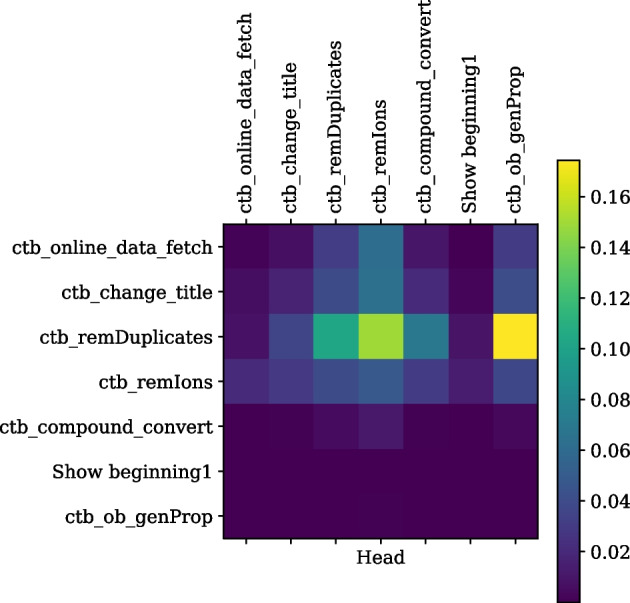
The self-attention weights for a tool sequence, read from left to right on the horizontal axis or top to bottom on the vertical axis, from the tool suite ChemicalToolBox (CTB) [42] in Galaxy Europe are shown. It is seen that tools from the CTB suite (containing “ctb” prefix) attend to each other as they have higher correlation weights, but they don’t attend to the “show beginning1” tool, which is only a text formatting tool and not from the CTB suite

**Fig. 5:**
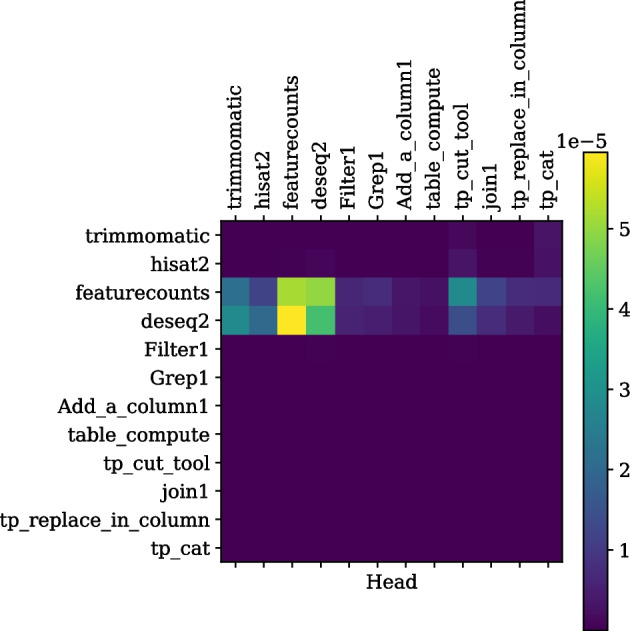
The self-attention weights for a tool sequence, read from left to right on the horizontal axis or top to bottom on the vertical axis and used for performing differential expression analysis, are shown. It is seen that tools such as trimmomatic [45], hisat2 [46], featurecounts [47] and deseq2 [48] are more correlated to each other compared to the text formatting tools such as “join1” or “filter1”

### Self-attention weights

As described in the Self-attention section, this technique is used in this work to learn vector representations of sequences of tools combining information from different parts of sequences. Self-attention weights measure the magnitude of relatedness for all pairs of tools in each tool sequence. It is represented by a matrix of size NxN, where N is the maximum length of a tool sequence (25). Two such matrices are computed by the trained transformer model for two tool sequences in the test dataset and plotted as shown in Figs. 4 and 5. The following two examples provide insights into the model’s internal mechanism. The first example in Fig. 4 has a workflow from the ChemicalToolBox (CTB) [42] tool suite. The pairs of tools from CTB such as “ctb_remIons” [42] and “ctb_remDuplicates” [42] (cell represented by light green colour in the third row of the fourth column) and “ctb_ob_genProp” [42] and “ctb_remDuplicates” [42] (cell represented by yellow colour in the third row of the seventh column) show higher attention weights than all other the CTB tools with the “show beginning1” tool, a general-purpose text extraction tool in Galaxy. The “show beginning1” tool does not correlate with CTB tools in the workflow, as depicted by extremely low attention weights (represented by deep violet colour). Another example in Fig. 5 shows a differential expression analysis workflow [43, 44] containing tools such as “trimmomatic” [45], “hisat2” [46], “featurecounts” [47], and “deseq2” [48]. These tools are often used together and correlate more than the other workflow tools. Tools such as “filter1” (used for filtering datasets), “grep1” (used for searching in datasets), and “join1” (used for joining two or more datasets) are text formatting tools that bear little correlation to the tools such as “trimmomatic”, “hisat2”, “featurecounts” and “deseq2” which are primarily used for analysing genomic sequences. These two examples show that the transformer model computes the sequence representation by learning the context from different sections of a tool sequence. These representations of tool sequences are further used for mapping to their respective multi-hot encoded labels. More examples of self-attention weight plots belonging to two scientific workflows are available in Additional files 5 and 6.

## Conclusion and discussion

Transformer architecture provides a robust way to model sequential data, such as linear sequences of tools extracted from Galaxy workflows and recommends tools with a high precision (precision@k) of approximately 0.98. It outperforms RNN, CNN and DNN for creating and using a tool recommendation model on multiple key indicators, such as faster computation of recommendations and model convergence. In addition, the transformer model generalises beyond the seen data to recommend higher-quality tools than all other models. Additionally, the transformer achieves good prediction accuracy for infrequent tools. These features will benefit researchers in exploring high-quality tools faster and promote exploratory data analysis in Galaxy. New models can be readily created in Galaxy and deployed using the recommendation model-creating tool. The proposed approach can be used with workflows created outside of Galaxy to create a recommendation system. However, the data pre-processing methods may need to be adapted.

### Trained model

The trained model, created in our work, combines trained weight matrices belonging to different layers of the transformer, Python dictionaries of mapping of tools with their respective indices and other utility files to create an HDF5 model file [49]. The composite HDF5 file reconstructs the model while recommending tools using an API [50] in Galaxy Europe. Alternatively, the transformer model can be easily created on Galaxy Europe by running a tool [51] that executes the same scripts as available in the codebase [26]. This tool needs Galaxy workflows and tool usage files as inputs. Steps to create and deploy such a model on any Galaxy server are written in the “README” file [26].

### Post processing

The usage of the tool recommendation system can be explored on Galaxy Europe. The API [50] considers the current sequence of tools from the workflow editor in Galaxy Europe or any tool for showing recommendations (Additional files 7 and 8). The input tool or a tool sequence is read by the trained model [49] to predict tools. These predicted tools may contain a few invalid tools that should not be used to extend the current workflow. In the post-processing step of the predicted tools, recommendations are refined to remove such tools from the predicted list of tools with incompatible datatypes that are unavailable or deprecated from Galaxy Europe. In addition, the refined list is then sorted in descending order of their usage in the last year in Galaxy Europe. It enables the highly used tool in the past year to appear at the top of the recommendations.

### Recommended tools in Galaxy

After refining the recommendations made by the trained transformer model in the post-processing step, the refined list of predicted tools is shown in Galaxy as two user interface (UI) elements - one after running a tool that shows recommended tools as branches of a tree and the second in the Galaxy workflow editor inside a tool’s box. The right arrow on the top right corner inside a tool’s box shows a list of recommended tools. UI elements used in Galaxy for showing tool recommendations are shown in Additional files 7 and 8.

## Future work

To improve the tool recommendation model’s generalisation performance, ways to integrate each tool’s metadata in training workflows may be explored. Transformer provides a robust architecture to learn high-quality sequential representations. It may allow the model to improve the grouping of similar tools as recommendations. Another way to use a transformer is as a sequence-to-sequence model in which a part of a workflow is used as an input sequence and the remaining part as the output sequence. It could provide insights into how many ways a tool or a sequence of tools can be used by recommending multiple future tool sequences. Exploring and utilising its potential for solving more complex biological problems, such as the prediction of post-translational modification (PTM) sites such as de-phosphorylation by learning to embed amino-acids and protein sequences and drug-target interaction prediction would be highly beneficial to the scientific community.

## Availability and requirements

Project name: Tool recommender system in Galaxy using Transformers. Project home page: https://github.com/anuprulez/galaxy_tool_recommendation_transformers. Operating system(s): Linux. Programming language: Python, XML. Other requirements: TensorFlow. License: MIT License. Any restrictions to use by non-academics: None

### Supplementary Information

**Additional file 1. **A sample Galaxy workflow for machine learning analysis consisting of several individual tools.

**Additional file 2. **A workflow with 3 tools (Tool A, Tool B and Tool C) is represented as a sequence of integers shown in step B. The set of recommended tools is shown in step C, represented as a one-hot encoded vector shown in step D.

**Additional file 3. **Comparison of the precision@k metric for the transformer, RNN, CNN and DNN models for all tools (solid lines) and the lowest 25% of tools (dotted lines).

**Additional file 4. **Comparison of tool recommendations (top 20) by different neural network models for various scientific analyses/workflows.

**Additional file 5. **Attention weights of a workflow from HiC analyses shown as a heatmap.

**Additional file 6. **Attention weights for "Kc-align" and "Sarscov2formatter" tools along with the text formatting tools such as "cut1" and "remove beginning1".

**Additional file 7. **Recommended tools for the "Fastp" tool are shown as a list in Galaxy's workflow editor.

**Additional file 8. **Recommended tools for the BWA-MEM tool shown as leaves of a tree.

## Data Availability

All input data, scripts and models can be found at https://github.com/anuprulez/galaxy_tool_recommendation_transformers. All datasets containing workflows and recent usage of tools can be found at https://doi.org/10.5281/zenodo.7825973. A Jupyter notebook is created at https://github.com/anuprulez/galaxy_tool_recommendation_transformers/blob/master/notebooks/evaluate_model.ipynb to analyse predictions by the RNN and transformer models

## Abbreviations

AUC: Area under the receiver operating characteristic (ROC) curve
GB: Gigabyte
AI: Artificial intelligence
RNN: Recurrent neural network
CNN: Convolutional neural network
DNN: Dense neural network
PTM: Post-translational modification
UI: User interface
BERT: Bidirectional encoder representations from transformers
WINGS: Workflow instance generation and specialisation
PROPHETS: Process realisation and optimisation platform using human-readable expression of temporal-logic synthesis
