# Supplementary material for: Transformer-based tool recommendation system in Galaxy
Source: BMC Bioinformatics. 2023 Nov 27;24:446. doi: 10.1186/s12859-023-05573-w (PMC10680333; doi:10.1186/s12859-023-05573-w)
Supplement: Supplementary file 1 — Additional file 1. A sample Galaxy workflow for machine learning analysis consisting of several individual tools. [file 12859_2023_5573_MOESM1_ESM.pdf]

Transformer-based tool recommendation system in Galaxy

Anup Kumar[1,*], Bj¨orn Gr¨uning[1], Rolf Backofen[1,2]

[1] Bioinformatics Group, Department of Computer Science, University of Freiburg, Georges-Koehler-Allee 106, 79110 Freiburg, Germany

[2] Signalling Research Centres BIOSS and CIBSS, University of Freiburg, Schaenzlestr. 18, 79104 Freiburg, Germany

Bioinformatics Group, Department of Computer Science, University of Freiburg, Georges-Koehler-Allee 106, 79110 Freiburg, Germany
* kumara@informatik.uni-freiburg.de

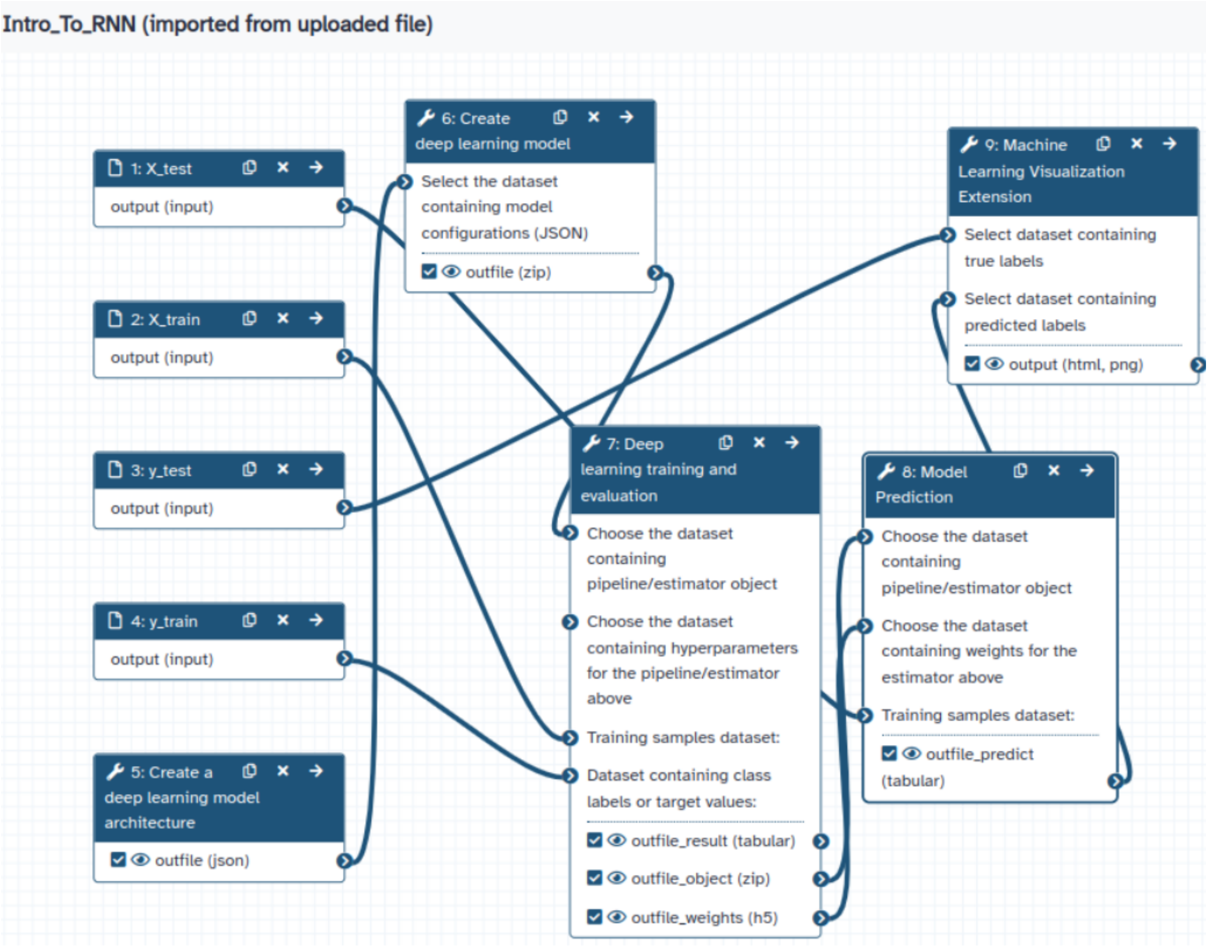

The figure shows a sample Galaxy workflow for machine learning consisting of several individual tools. It is executed as one unit of the software after attaching it with the necessary datasets. Approximately 60,000 such workflows are utilised in our work to create a tool recommendation model.
