# Supplementary material for: Transformer-based tool recommendation system in Galaxy
Source: BMC Bioinformatics. 2023 Nov 27;24:446. doi: 10.1186/s12859-023-05573-w (PMC10680333; doi:10.1186/s12859-023-05573-w)
Supplement: Supplementary file 2 — Additional file 2. A workflow with 3 tools (Tool A, Tool B and Tool C) is represented as a sequence of integers shown in step B. The set of recommended tools is shown in step C, represented as a one-hot encoded vector shown in step D. [file 12859_2023_5573_MOESM2_ESM.pdf]

Transformer-based tool recommendation system in Galaxy

Anup Kumar[1,*], Björn Grüning[1], Rolf Backofen[1,2]

[1] Bioinformatics Group, Department of Computer Science, University of Freiburg, Georges-Koehler-Allee 106, 79110 Freiburg, Germany

[2] Signalling Research Centres BIOSS and CIBSS, University of Freiburg, Schaenzlestr. 18, 79104 Freiburg, Germany

Bioinformatics Group, Department of Computer Science, University of Freiburg, Georges-Koehler-Allee 106, 79110 Freiburg, Germany
* kumara@informatik.uni-freiburg.de

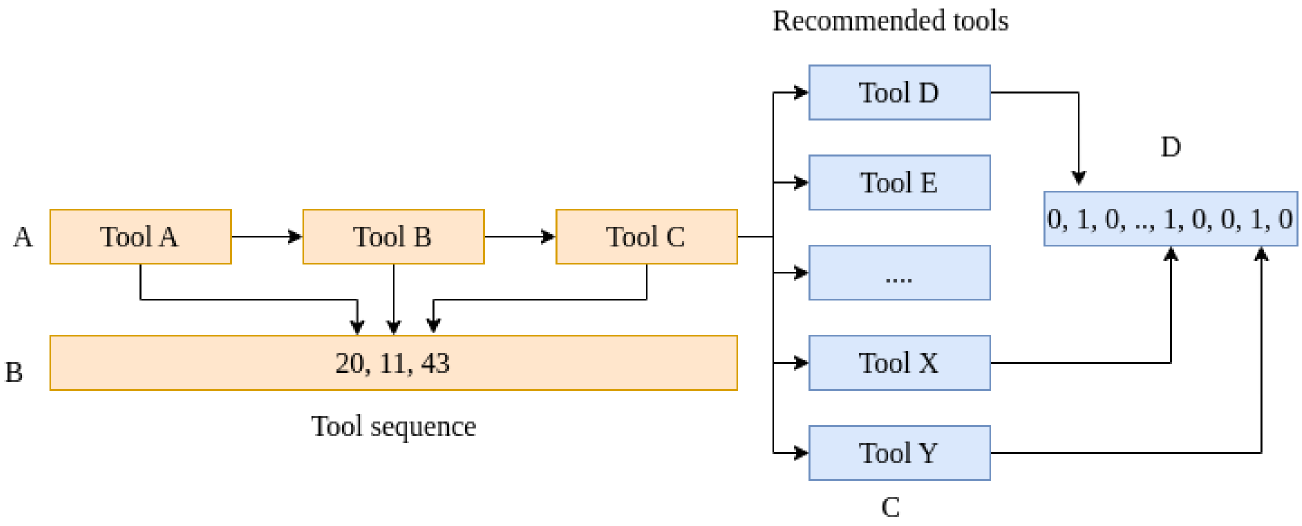

The figure shows a sample workflow with 3 tools (Tool A, Tool B and Tool C). This workflow is represented as a sequence of integers shown in step B. The set of recommended tools is shown in step C which is represented as a one-hot encoded vector shown in step D. The position corresponding to the index of a tool is set to 1. Other positions remain 0. The corresponding pairs of B and D are used to create training and test datasets. The training dataset is used for training the transformer, RNN, CNN, and DNN models and the test dataset is used for evaluating the models.
