# Supplementary material for: Transformer-based tool recommendation system in Galaxy
Source: BMC Bioinformatics. 2023 Nov 27;24:446. doi: 10.1186/s12859-023-05573-w (PMC10680333; doi:10.1186/s12859-023-05573-w)
Supplement: Supplementary file 3 — Additional file 3. Comparison of the precision@k metric for the transformer, RNN, CNN and DNN models for all tools (solid lines) and the lowest 25% of tools (dotted lines). [file 12859_2023_5573_MOESM3_ESM.pdf]

Transformer-based tool recommendation system in Galaxy

Anup Kumar[1,*], Bj¨orn Gr¨uning[1], Rolf Backofen[1,2]

[1] Bioinformatics Group, Department of Computer Science, University of Freiburg, Georges-Koehler-Allee 106, 79110 Freiburg, Germany

[2] Signalling Research Centres BIOSS and CIBSS, University of Freiburg, Schaenzlestr. 18, 79104 Freiburg, Germany

Bioinformatics Group, Department of Computer Science, University of Freiburg, Georges-Koehler-Allee 106, 79110 Freiburg, Germany
* kumara@informatik.uni-freiburg.de

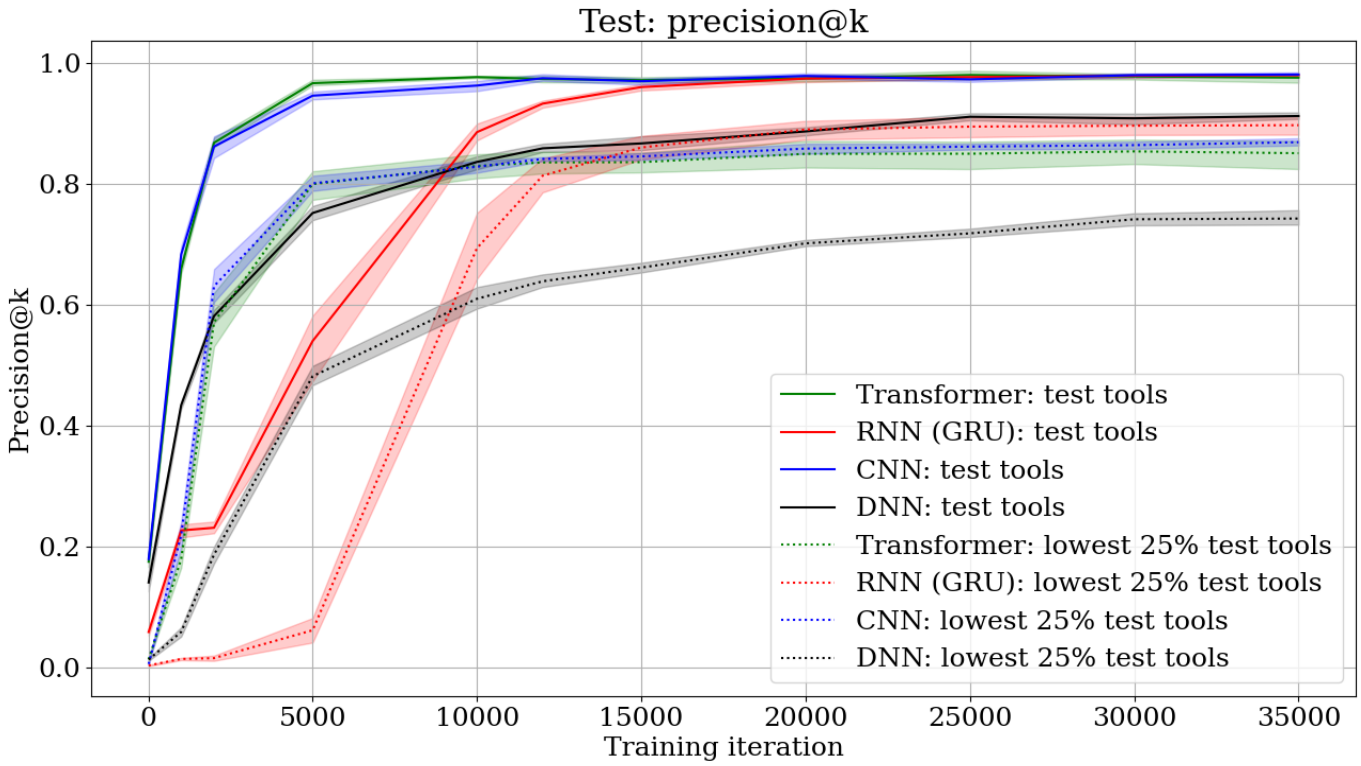

The image compares the precision@k metric for the transformer, RNN, CNN and DNN models for all tools (solid lines) and the lowest 25% of tools (dotted lines). The transformer, RNN and CNN models achieve similar precision@k metric values for all tools (approximately 0.98) but at different training iterations. The transformer converges (slightly before 10,000) to precision@k = 0.98 slightly earlier than CNN (at around 12,000) and relatively earlier than RNN (at around 20,000). The transformer model achieves a similar precision@k score for infrequent tools (the lowest 25% of all tools used for creating the workflows) compared to the CNN model (approximately 0.85), and it is slightly worse

than RNN. DNN performs worst of all models compared and achieves precision@k value of 0.9 for all tools and 0.75 for infrequent tools.
