# Supplementary material for: Transformer-based tool recommendation system in Galaxy
Source: BMC Bioinformatics. 2023 Nov 27;24:446. doi: 10.1186/s12859-023-05573-w (PMC10680333; doi:10.1186/s12859-023-05573-w)
Supplement: Supplementary file 4 — Additional file 4. Comparison of tool recommendations (top 20) by different neural network models for various scientific analyses/workflows. [file 12859_2023_5573_MOESM4_ESM.pdf]

Transformer-based tool recommendation system in Galaxy

Anup Kumar[1,*], Bj ̈orn Gr ̈uning[1], Rolf Backofen[1,2]

[1] Bioinformatics Group, Department of Computer Science, University of Freiburg, Georges-Koehler-Allee 106, 79110 Freiburg, Germany

[2] Signalling Research Centres BIOSS and CIBSS, University of Freiburg, Schaenzlestr. 18, 79104 Freiburg, Germany

Bioinformatics Group, Department of Computer Science, University of Freiburg, Georges-Koehler-Allee 106, 79110 Freiburg, Germany
* kumara@informatik.uni-freiburg.de

Additional Table 1: Comparison of tool recommendations (top 20) by different neural network architectures for different scientific analyses/workflows

Scientific analysis	Tool/tool sequence	Ground truth	Transformer	RNN	CNN	DNN
Variant analysis	snpeff_sars_cov_2	snpSift filter, snpSift extractFields, lofreq filter, vcf2tsv, multiqc, collapse dataset, CONVERTER vcf to vcf bgzip 0	multiqc, collapse dataset, CONVERTER vcf to vcf bgzip 0, snpSift extractFields, vcf2tsv, snpSift filter, lofreq filter, **freebayes, mimodd varcall, snpfreqplot, gemini load, vcfcombine**	multiqc, collapse dataset, vcf2tsv, CONVERTER vcf to vcf bgzip 0, lofreq filter, snpSift extractFields, snpSift filter, **tb variant filter, mimodd map, vcffilter2**	snpSift_filter, collapse_dataset, CONVERTER_vcf_to_vcf_bgzip_0, snpSift_extractFields, lofreq_filter, multiqc, vcf2tsv, **gemini_annotate**, **gemini_load**, **bedtools_intersectbed**	collapse_dataset, multiqc, **tb_variant_filter**
Single-cell	anndata_import	scanpy filter, anndata inspect, anndata manipulate, ucsc cell browser, scanpy inspect, scanpy filter cells	scanpy filter cells, anndata inspect, ucsc cell browser, scanpy filter, scanpy inspect, anndata manipulate, **scanpy normalise data, scanpy plot, anndata ops, scanpy remove confounders, scanpy integrate**	anndata manipulate, scanpy filter, scanpy filter cells, ucsc cell browser, scanpy inspect, anndata inspect, **scanpy plot, scanpy normalise data, scmap scmap cluster, scmap scmap cell,**	scanpy_filter_cells,anndata_manipulate,scanpy_inspect,anndata_inspect,scanpy_filter, ucsc_cell_browser, **scanpy_integrate_harmony, scanpy_remove_confounders, scanpy_cluster_reduce_dimension, sccaf_regress_out,**	anndata_inspect, scanpy_filter_cells, ucsc_cell_browser, scanpy_filter, scanpy_inspect, anndata_manipulate, **scanpy_normalise_data, scanpy_plot, anndata_ops, scanpy_run_tsne, scanpy_integrate_harmony,**

			**harmony, scanpy normalize, scpred get feature space, scanpy find variable genes, scpred predict labels, scpred eigen decompose**	**scanpy filter genes**	**scanpy_plot, scanpy_compute_graph, scanpy_run_tsne, anndata_ops**	**scanpy_normalize, scanpy_plot_embed, scanpy_remove_confounders, scanpy_plot_trajectory, scanpy_compute_graph, scanpy_filter_genes, scanpy_find_markers**
Deep learning	keras train and eval	model prediction, ml visualization ex	model prediction, ml visualization ex, **plotly regression-performance plot, sklearn discriminant classifier, plotly ml performance plots**	model prediction, ml visualization ex, **nn classifier**	model_prediction, ml_visualization_ex	ml_visualization_ex, model_prediction
Proteomics	mass spectrometry-imaging filtering, cardinal-preprocessing, cardinal-segmentations	Filter1	Filter1, **cardinal spectra plots, cardinal combine, cardinal mz images, cardinal classification, cardinal data exporter, cardinal quality report, maldi quant preprocessing, cardinal preprocessing, cardinal segmentations, cardinal filtering, mass spectrometry-imaging filtering, maldi quant peak detection**	Filter1	Filter1, **cardinal_classification, cardinal_preprocessing, cardinal_data_exporter, cardinal_quality_report, cardinal_segmentations, cardinal_mz_images**	**cardinal_spectra_plots, cardinal_preprocessing, cardinal_quality_report, cardinal_mz_images, cardinal_classification, cardinal_combine, cardinal_filtering, cardinal_data_exporter, cardinal_segmentations, maldi_quant_peak_detection**

The above table compares tool recommendations by different architectures of neural networks. The transformer, RNN, CNN and DNN architectures have been trained on the same training dataset (sequence of tools) for over 35,000 iterations. In each iteration, 512 (batch size) tool sequences are selected for training following uniform sampling to mitigate overfitting on dominant sequences of tools and improve prediction accuracy on tool sequences containing infrequent tools. Parameters of individual networks can be found in the manuscript and the code repository.

For each trained model, the top 20 tool recommendations are predicted and compared to the ground truth and with one another. The tools shown in bold are those recommended tools not seen during training - not present in the set of ground truth tools for the tool/tool sequence. From the list of top 20 recommendations, invalid or sub-optimal tools are removed to keep only the relevant tools.

Variant calling:

In the first row of the above table, tools are recommended using different models for the "snpeff_sars_cov_2" tool. The recommended tools (top 20) by the transformer, RNN and CNN models contain all the tools in the ground truth tools, but DNN predicts only 2 out of 7 tools in the ground truth. In addition, the transformer, RNN, and CNN models predict a few other tools that belong to the same field, variant calling, of bioinformatic analyses, which were unseen during training. Transformer recommends 5 tools beyond training data, while RNN and CNN could predict only three each. However, DNN could predict only one such tool. Transformer recommends more relevant tools than RNN, CNN, and DNN for the "snpeff_sars_cov_2" tool.

Single-cell:

In the second row, recommendations of the "anndata_import" tool by all four models are shown. All the models predict all the tools in ground truth correctly. Surprisingly, DNN recommends the most extensive set of tools (12 out of 20 predicted) going beyond training data, while transformer, RNN and DNN recommended only 10, 5 and 8, respectively. Although DNN outperforms other models for the "anndata_import" tool, the transformer still produces relevant recommendations and stays close to the performance of DNN.

Deep learning:

In the third row, recommendations of the "keras train and eval" tool by all four models are shown. All models correctly predict the ground truth tools. In addition to correctly predicting ground truth tools, the transformer recommends three additional tools relevant to any machine learning analysis. RNN could recommend only one such tool, and CNN and DNN fail to recommend any such tool. Therefore, the transformer outperforms all other models for recommending "keras train and eval" tools.

Proteomics:

Transformer, RNN, and CNN models correctly predict the only ground truth tool, but DNN fails to predict it. Instead of recommending tools for a tool, a tool sequence's "mass spectrometry-imaging filtering => cardinal-preprocessing => cardinal-segmentations" recommendations are shown in the fourth row. However, DNN recommends ten such tools that are relevant and beyond training data, while CNN predicts only six such tools, and RNN fails to predict any such tool. However, they are outperformed by the transformer recommending 12 tools beyond training data.

By analysing and comparing the recommendations (combination of the ground truth and relevant tools unseen during training) made by all models (transformer, RNN, CNN and DNN), it is concluded that the transformer model outperforms RNN, CNN, and DNN models in recommending high-quality tools for various scientific analyses.
