# Supplementary material for: Transformer-based tool recommendation system in Galaxy
Source: BMC Bioinformatics. 2023 Nov 27;24:446. doi: 10.1186/s12859-023-05573-w (PMC10680333; doi:10.1186/s12859-023-05573-w)
Supplement: Supplementary file 5 — Additional file 5. Attention weights of a workflow from HiC analyses shown as a heatmap. [file 12859_2023_5573_MOESM5_ESM.pdf]

Transformer-based tool recommendation system in Galaxy

Anup Kumar[1,*], Björn Grüning[1], Rolf Backofen[1,2]

[1] Bioinformatics Group, Department of Computer Science, University of Freiburg, Georges-Koehler-Allee 106, 79110 Freiburg, Germany

[2] Signalling Research Centres BIOSS and CIBSS, University of Freiburg, Schaenzlestr. 18, 79104 Freiburg, Germany

Bioinformatics Group, Department of Computer Science, University of Freiburg, Georges-Koehler-Allee 106, 79110 Freiburg, Germany

* kumara@informatik.uni-freiburg.de

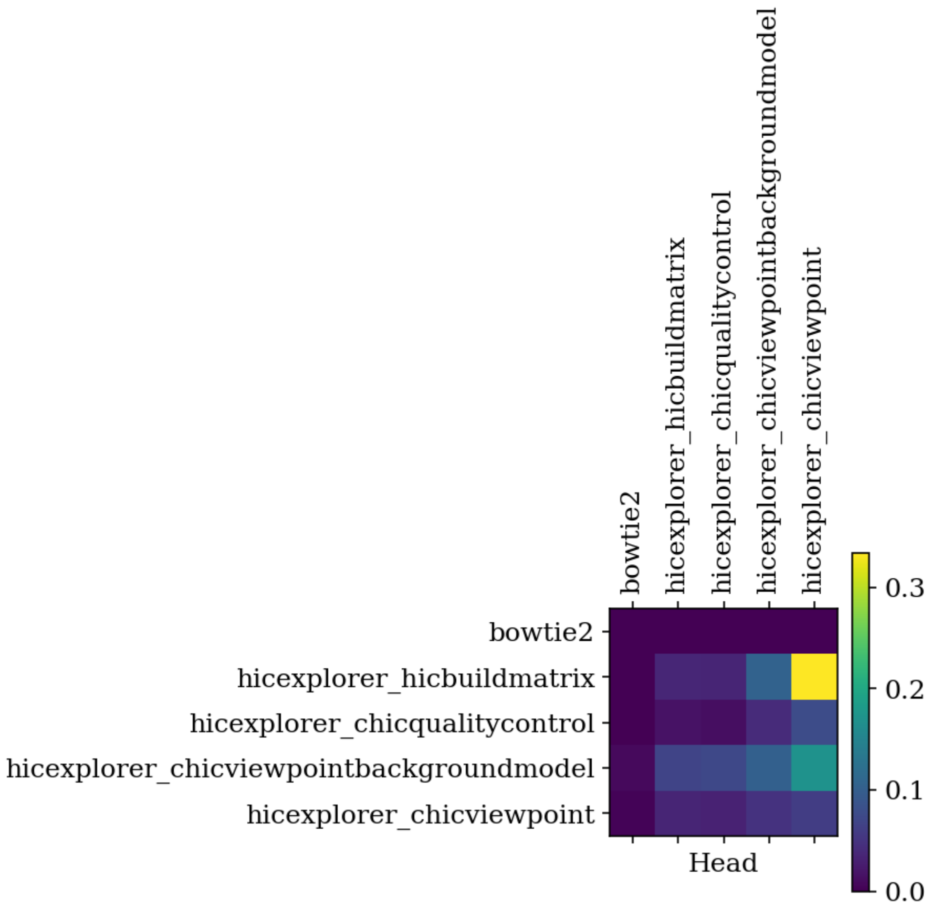

The figure shows attention weights of a workflow from HiC analyses. Attention weights are higher (shown by yellow and green colours) for the HiCExplorer tools (containing "hicexplorer" prefix) that appear together than those with "bowtie2" (shown by violet colour).
