# Supplementary material for: Transformer-based tool recommendation system in Galaxy
Source: BMC Bioinformatics. 2023 Nov 27;24:446. doi: 10.1186/s12859-023-05573-w (PMC10680333; doi:10.1186/s12859-023-05573-w)
Supplement: Supplementary file 6 — Additional file 6. Attention weights for "Kc-align" and "Sarscov2formatter" tools along with the text formatting tools such as "cut1" and "remove beginning1". [file 12859_2023_5573_MOESM6_ESM.pdf]

Transformer-based tool recommendation system in Galaxy

Anup Kumar[1,*], Bj¨orn Gr¨uning[1], Rolf Backofen[1,2]

[1] Bioinformatics Group, Department of Computer Science, University of Freiburg, Georges-Koehler-Allee 106, 79110 Freiburg, Germany

[2] Signalling Research Centres BIOSS and CIBSS, University of Freiburg, Schaenzlestr. 18, 79104 Freiburg, Germany

Bioinformatics Group, Department of Computer Science, University of Freiburg, Georges-Koehler-Allee 106, 79110 Freiburg, Germany
* kumara@informatik.uni-freiburg.de

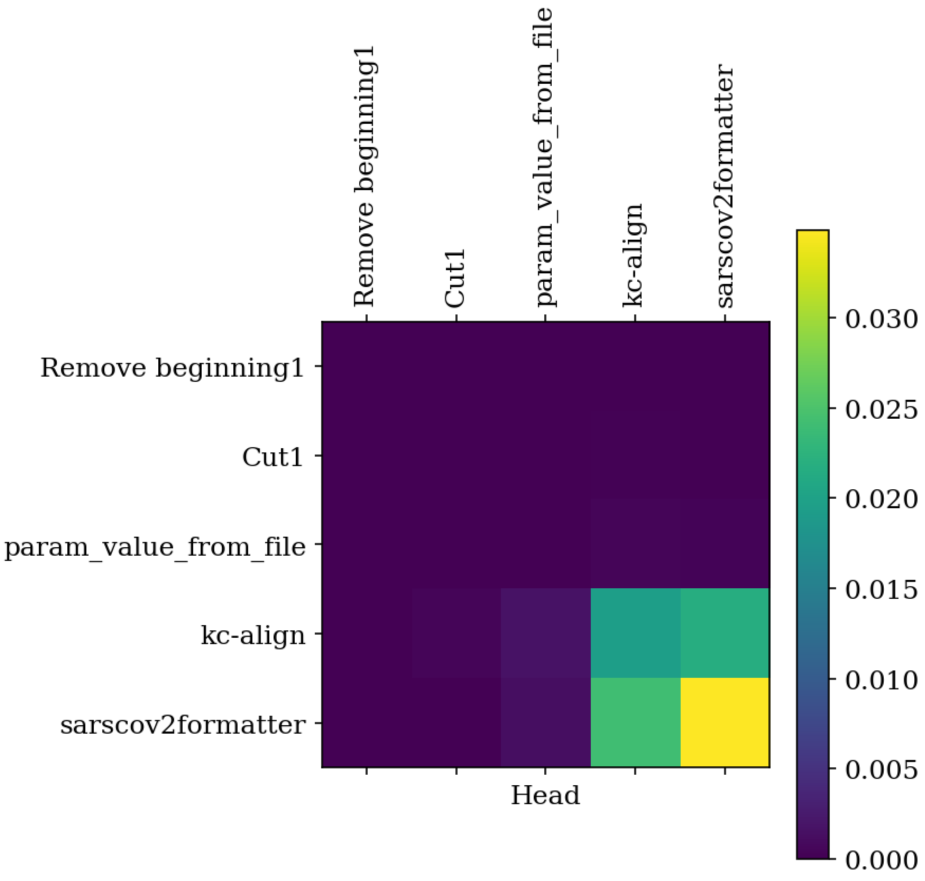

The figure shows attention weights for "kc-align" and "sarscov2formatter" tools along with the text formatting tools such as "cut1" and "remove beginning1". Attention weights are higher between "kc-align" and "sarscov2formatter" tools than those tools with the text formatting tools such as "remove beginning1" and "cut1".
