# Supplementary material for: Transformer-based tool recommendation system in Galaxy
Source: BMC Bioinformatics. 2023 Nov 27;24:446. doi: 10.1186/s12859-023-05573-w (PMC10680333; doi:10.1186/s12859-023-05573-w)
Supplement: Supplementary file 7 — Additional file 7. Recommended tools for the "Fastp" tool are shown as a list in Galaxy's workflow editor. [file 12859_2023_5573_MOESM7_ESM.pdf]

Transformer-based tool recommendation system in Galaxy

Anup Kumar[1,*], Bj ̈orn Gr ̈uning[1], Rolf Backofen[1,2]

[1] Bioinformatics Group, Department of Computer Science, University of Freiburg, Georges-Koehler-Allee 106, 79110 Freiburg, Germany

[2] Signalling Research Centres BIOSS and CIBSS, University of Freiburg, Schaenzlestr. 18, 79104 Freiburg, Germany

Bioinformatics Group, Department of Computer Science, University of Freiburg, Georges-Koehler-Allee 106, 79110 Freiburg, Germany
* kumara@informatik.uni-freiburg.de

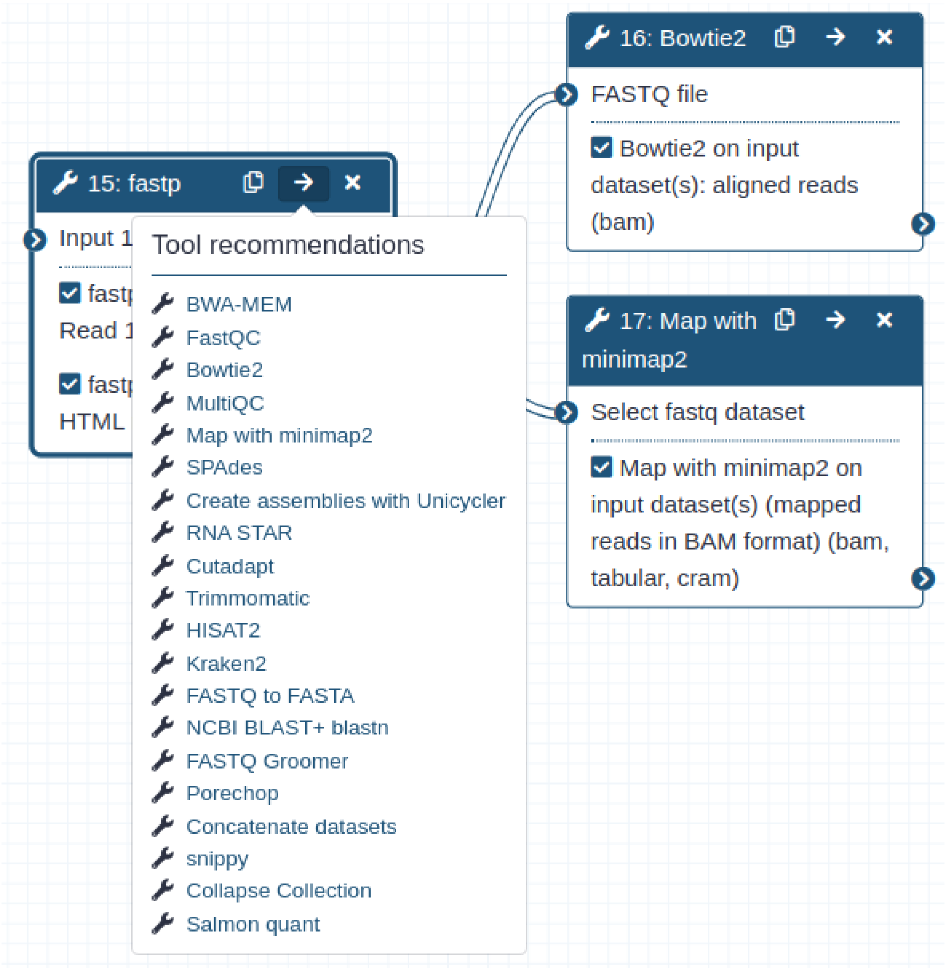

The image shows recommended tools for the "Fastp" tool. Two recommended mapping tools, "Bowtie2" and "Map with minimap2" are shown too. It is a part of the workflow for Unicycler assembly of SARS-CoV-2 genome with preprocessing to remove human genome reads.
