# Supplementary material for: Transformer-based tool recommendation system in Galaxy
Source: BMC Bioinformatics. 2023 Nov 27;24:446. doi: 10.1186/s12859-023-05573-w (PMC10680333; doi:10.1186/s12859-023-05573-w)
Supplement: Supplementary file 8 — Additional file 8. Recommended tools for the BWA-MEM tool shown as leaves of a tree. [file 12859_2023_5573_MOESM8_ESM.pdf]

Transformer-based tool recommendation system in Galaxy

Anup Kumar[1,*], Björn Grüning[1], Rolf Backofen[1,2]

[1] Bioinformatics Group, Department of Computer Science, University of Freiburg, Georges-Koehler-Allee 106, 79110 Freiburg, Germany

[2] Signalling Research Centres BIOSS and CIBSS, University of Freiburg, Schaenzlestr. 18, 79104 Freiburg, Germany

Bioinformatics Group, Department of Computer Science, University of Freiburg, Georges-Koehler-Allee 106, 79110 Freiburg, Germany
* kumara@informatik.uni-freiburg.de

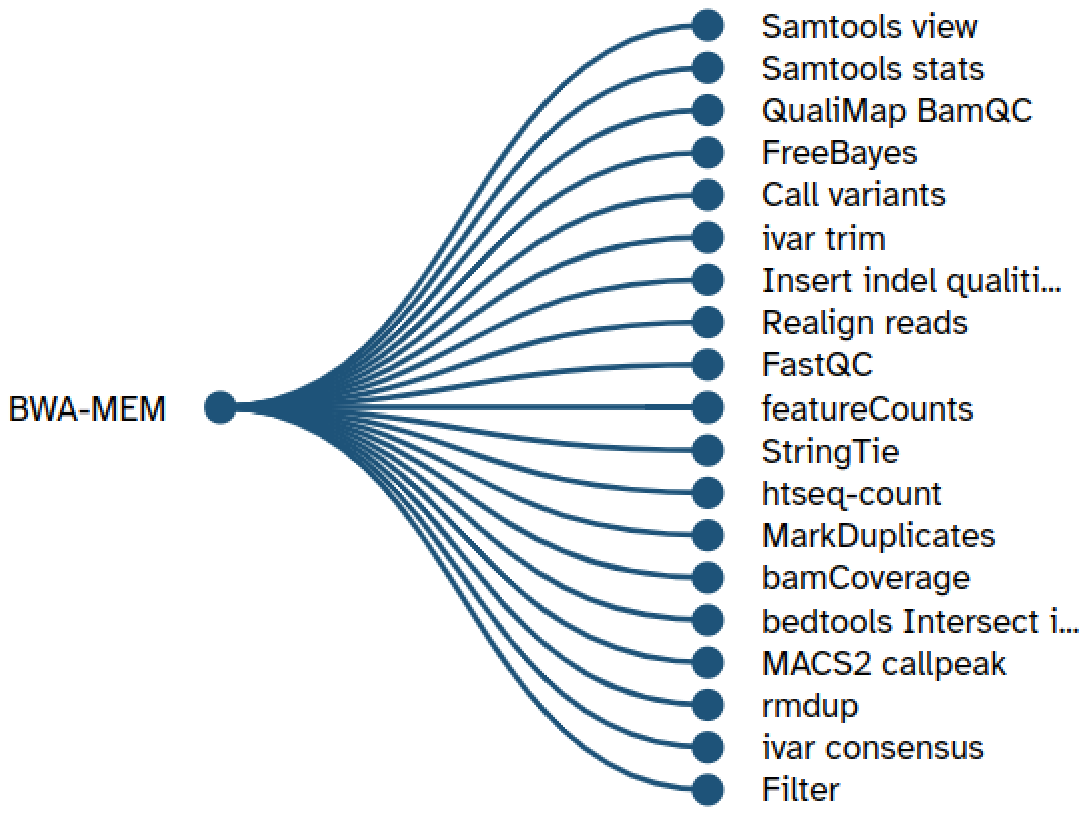

The figure shows tool recommendations for tool BWA-MEM (mapper software tool in Galaxy) as leaves of the tree. These recommended tools can be applied to the datasets such as a ".bam" file produced by the BWA-MEM tool.
